# Discriminating the eight genotypes of the porcine circovirus type 2 with TaqMan-based real-time PCR

**DOI:** 10.1186/s12985-021-01541-z

**Published:** 2021-04-07

**Authors:** Ellen Kathrin Link, Matthias Eddicks, Liangliang Nan, Mathias Ritzmann, Gerd Sutter, Robert Fux

**Affiliations:** 1 Division of Virology, Department of Veterinary Sciences, LMU Munich, Veterinärstrasse 13, 80539 Munich, Germany; 2 Clinic for Swine at the Centre for Clinical Veterinary Medicine, LMU Munich, Sonnenstrasse 16, 85764 Oberschleissheim, Germany

**Keywords:** PCV2, Genotype, Real-time PCR, QPCR, TaqMan PCR, Locked nucleic acid

## Abstract

**Background:**

The porcine circovirus type 2 (PCV2) is divided into eight genotypes including the previously described genotypes PCV2a to PCV2f and the two new genotypes PCV2g and PCV2h. PCV2 genotyping has become an important task in molecular epidemiology and to advance research on the prophylaxis and pathogenesis of PCV2 associated diseases. Standard genotyping of PCV2 is based on the sequencing of the viral genome or at least of the open reading frame 2. Although, the circovirus genome is small, classical sequencing is time consuming, expensive, less sensitive and less compatible with mass testing compared with modern real-time PCR assays. Here we report about a new PCV2 genotyping method using qPCR.

**Methods:**

Based on the analysis of several hundred PCV2 full genome sequences, we identified PCV2 genotype specific sequences or single-nucleotide polymorphisms. We designed six TaqMan PCR assays that are specific for single genotypes PCV2a to PCV2f and two qPCRs targeting two genotypes simultaneously (PCV2g/PCV2d and PCV2h/PCV2c). To improve specific binding of oligonucleotide primers and TaqMan probes, we used locked nucleic acid technology. We evaluated amplification efficiency, diagnostic sensitivity and tested assay specificity for the respective genotypes.

**Results:**

All eight PCV2 genotype specific qPCRs demonstrated appropriate amplification efficiencies between 91 and 97%. Testing samples from an epidemiological field study demonstrated a diagnostic sensitivity of the respective genotype specific qPCR that was comparable to a highly sensitive pan-PCV2 qPCR system. Genotype specificity of most qPCRs was excellent. Limited unspecific signals were obtained when a high viral load of PCV2b was tested with qPCRs targeting PCV2d or PCV2g. The same was true for the PCV2a specific qPCR when high copy numbers of PCV2d were tested. The qPCR targeting PCV2h/PCV2c showed some minor cross-reaction with PCV2d, PCV2f and PCV2g.

**Conclusion:**

Genotyping of PCV2 is important for routine diagnosis as well as for epidemiological studies. The introduced genotyping qPCR system is ideal for mass testing and should be a valuable complement to PCV2 sequencing, especially in the case of simultaneous infections with multiple PCV2 genotypes, subclinically infected animals or research studies that require large sample numbers.

## Background

One of the interesting features of the porcine circovirus type 2 (PCV2), a member of the family Circoviridae within the realm Monodnaviria, is the high nucleotide substitution rate, which leads to an evolutionary dynamic that is comparable to that of RNA viruses and lesser to that of double stranded DNA viruses [1]. Recent studies, analyzing appropriate big data sets, estimated the rate at 1.4 × 10^–3^ to 7.8 × 10^–4^ substitutions per site per year [2], with a higher rate for the genotype PCV2a compared to PCV2b and PCV2d, and a slightly higher rate for the open reading frame 2 (ORF2; capsid protein gene) compared to ORF1 (replicase protein gene) [3]. Although PCV2 has a very small genome (1767–1777 bp), the high evolutionary rate is the cause of a great abundance of PCV2 variants in the field. Therefore, the precise classification of strains within the species PCV2 has been the source for controversial discussions. Recently, Franzo and Segales proposed a new genotyping methodology based on a phylogeny-grounded genotype definition including three requirements: (1) a maximum intra-genotype p-distance of the ORF2 sequence of 13%, (2) a bootstrap support at the corresponding internal node > 70% and (3) at least fifteen available sequences. This classification scheme allowed the definition of eight PCV2 genotypes, including the previously described genotypes PCV2a to PCV2f and the two new genotypes PCV2g and PCV2h [4] (see Table 1).

**Table 1:** Typical features of PCV2 genotypes

Genotype	Full genome	ORF2	Occurrence
PCV2a	1768 bp	702 bp/233 aa	worldwide
PCV2b	1767 bp	702 bp/233 aa	worldwide
PCV2c	1767 bp	705 bp/234 aa	Brazil, China?, Denmark^a^
PCV2d	1767 bp	705 bp/234 aa	worldwide
PCV2e	1777 bp	717 bp/238 aa	China, Japan, Mexico, USA
PCV2f	1767 bp	705 bp/234 aa	Asia (China, India, Indonesia), Brazil, Croatia
PCV2g	1767 bp or 1768 bp	702 bp/233 aa or 705 bp/234 aa	Asia, Europe (Germany, Romania, Ukraine)
PCV2h	1767 bp	705 bp/234aa	Asia (China, India, Indonesia, Thailand, Viet Nam)

^a^Historical samples

Up to now, the earliest proof of PCV2 in swine was reported from a German sample from 1962 [5]. In this specimen, PCV2 detection was positive using DNA in situ hybridization and PCR. However, since only a 66 bp sequence of ORF1 (GenBank EU158775) is available in this case, the genotype cannot be specified. One can speculate whether this is the first evidence of PCV2a, as it is believed that this genotype is the first to circulate in the pig population worldwide and has been linked to the onset of the postweaning multisystemic wasting syndrome (PMWS) [6–9]. In the first period, PCV2a was the most prevalent PCV2 genotype in clinically affected pigs. This changed with the rise of a second cluster of PCV2 variants, eventually called PCV2b [10]. Initially in Europe, later in North America, an increased incidence of PCV2 associated diseases (PCVD) was observed in the early 2000s, this time caused by the genotype PCV2b, which replaced PCV2a as the dominant virus variant in PCVD outbreaks [8, 11–13]. This change is commonly known as “PCV2 genotype shift”. For several years PCV2b dominated the world as causative agent of PCVD [3, 14–16] and caused severe economic losses. In 2008, Dupont and colleagues published a retrospective study of PCV2 isolates from Danish archives. Within this collection, three genome sequences obtained from samples collected in 1980, 1987 and 1990 did not cluster with known PCV2a or 2b strains, but were assigned to a new branch of the PCV2 phylogenetic tree, later referred to as genotype PCV2c [8]. Interestingly, after their discovery, PCV2c strains were not detected in swine populations worldwide for a long period. More recently, PCV2c was detected in feral pigs in the Brazilian Pantanal [17] and a Chinese group reported potential recombinants between PCV2b and PCV2c strains [18]. However, for global swine industry PCV2c is still of minor interest today. In contrast, genotype PCV2d is a challenge and a serious economic problem in pig production systems all over the world. It has become the most prevalent PCV2 genotype (“second genotype shift”), at least in clinically affected animals. About a decade ago, Guo and colleagues reported an emerging “mutant” PCV2 (strain BDH, HM038017) characterized by a mutation in the stop codon of ORF2 resulting in a capsid protein of 234 amino acid (aa) and which showed higher levels of virulence compared to classical PCV2a or 2b strains [19, 20]. Remarkably, other groups could not confirm a particular virulence of this virus variant [21]. However, BDH-like viruses spread quickly to North America [22], Europe [23] and the rest of the world [24]. In the phylogenetic tree of PCV2, these viruses formed a separate cluster together with virus strains originally described by Olvera and colleagues as subgroup 1C (= PCV2b, subgroup 1C) [13]. Therefore, different names were used for this group for some time (mutant PCV2, mPCV2b, PCV2b-1C), but eventually this cluster was accepted as the genotype PCV2d [2]. Although the PCV2d reference strain BDH was isolated in 2008 [19], the oldest published PCV2d sequence (AY484410) was obtained from a Dutch study carried out in 2001/2002 [25]. Furthermore, Xiao and colleagues demonstrated a significant heterogeneity within the genotype PCV2d and subdivided it into the major clades PCV2d-1 (containing mainly “old” sequences, obtained before 2008) and PCV2d-2 (BDH-like strains) [24]. This classification scheme was used in several other studies [2, 26, 27] until the new genotyping methodology [4] transferred most of the PCV2d-1 strains into the new genotype PCV2g. Interestingly, some of the former PCV2d-1 members (e.g. AY484410 or AY181946) remained within in the “new” genotype 2d. Remarkably, most PCV2g strains have been isolated from wild boars. In 2016, Davies and colleagues reported a unique new cluster of PCV2 strains, collected in North America from 2006 to 2015. These viruses were characterized by a larger genome size due to an insertion at the 3′ end of ORF2, causing a 238 aa capsid protein (see Table 1). This cluster, named genotype PCV2e, showed the highest distance to all other PCV2 genotypes, and it was hypothesized that it might be a kind of PCV2 ancestor [28]. Meanwhile, PCV2e strains have also been detected in China [29] and Japan (GenBank number LC278353). Bao and colleagues introduced genotype PCV2f in 2018, containing mainly strains from China, India and Indonesia [30]. Two isolates that are an exception were reported from a wild boar in Croatia (HQ591381) and feral pigs in Brazil (KJ094600), originally described as PCV2a [17]. The latter is showing a comparative big distance to the other PCV2f members. As described above, genotype PCV2g includes strains previously described as PCV2d-1, having a capsid protein of 234 aa. However, the analysis of Franzo and Segales [4] shows that this group also contains PCV2 sequences with a 233 aa ORF2 product and a total length of 1768 bp, which is typical for PCV2a strains. This might explain, why some of these strains were described as “inter-genotype” recombinants before [31]. One of the oldest members of this new genotype, sequence AY713470, is a good example of how complex correct genotyping of PCV2 strains can be. This strain originated from a German wild boar, which was hunted in the winter of 2003/04. The longer capsid protein (234 aa) and the full genome length of 1767 bp of the virus were explained by a one nucleotide deletion at the end of ORF2 of a PCV2a virus [32]. In 2007, Olvera and colleagues classified this virus as a member of group 1C [13], and after the proposal of Segales and colleagues for PCV2 genotype definition and nomenclature [10] it was reclassified as PCV2b. Three years later it was integrated into a putative new genotype PCV2d [19], but alternative names (e.g. PCV2b-1C, mPCV2b) were still used [22, 23, 33] until the revision of PCV2 genotyping by Franzo and colleagues in 2015, which exemplified the classification to genotype PCV2d [2]. In the same year Xiao and colleagues introduced the virus as one of the PCV2d-1 reference strains [24], until recently it became one of the reference strains for genotype PCV2g [4]. Simultaneously with PCV2g, the new genotype PCV2h was documented. Up to now, only PCV2 sequences from Asia belong to this group [4]. Some of these strains were previously described as recombinant viruses or as “intermediate” strains [24, 34, 35].

From the beginning, scientists around the world tried to correlate different phylogenetic clusters or genotypes of PCV2 with characteristics such as virulence or clinical manifestation [8, 13, 36]. Although virulence-contributing features of the PCV2 genome or proteome are still poorly understood, correct PCV2 genotyping has become an important task, not only for answering scientific questions, but also in routine diagnostic work. Molecular epidemiology, the detection of emerging genotypes, tracing PCV2 spreading by international and national trade, the investigation of viral evolution and the evaluation of vaccine effectiveness and cross protection are only a few points, which illustrate the importance of correct PCV2 genotyping [2, 3, 37, 38]. Because of its small, circular genome, full genome sequencing of PCV2 is comparatively easy. Several protocols to amplify and sequence overlapping genome fragments have been published [8, 12, 25, 39]. In general, samples from animals with PCVD can be targeted this way because of the high viral load in these specimens. Receiving high quality sequencing results from subclinically infected pigs is more challenging. Several attempts to increase sensitivity by nested PCR or unspecific amplification of the circular DNA of circoviruses by rolling circling amplification (RCA) before PCR have been published [14, 40]. However, real-time PCR is a valuable option for faster, cheaper, more sensitive and more mass compatible genotyping in routine diagnostics. Two multiplex TaqMan-based qPCR assays for the differentiation of PCV2a and PCV2b were published already a decade ago [41, 42]. With the rise of PCV2d as new dominant genotype, these assays lost their importance, especially, as in one of the assays, cross reaction of the PCV2b specific TaqMan probe with PCV2d strains was observed [22].

In order to support the recently described new classification scheme [4] and to enable a fast and sensitive determination of the PCV2 genotypes, we here report the development and evaluation of PCV2 genotype specific TaqMan-PCR assays, covering all eight PCV2 genotypes.

## Methods

### Identification of PCV2 genotype specific nucleotide sequences and design of oligonucleotide primers and TaqMan probes

**Fig. 1:**
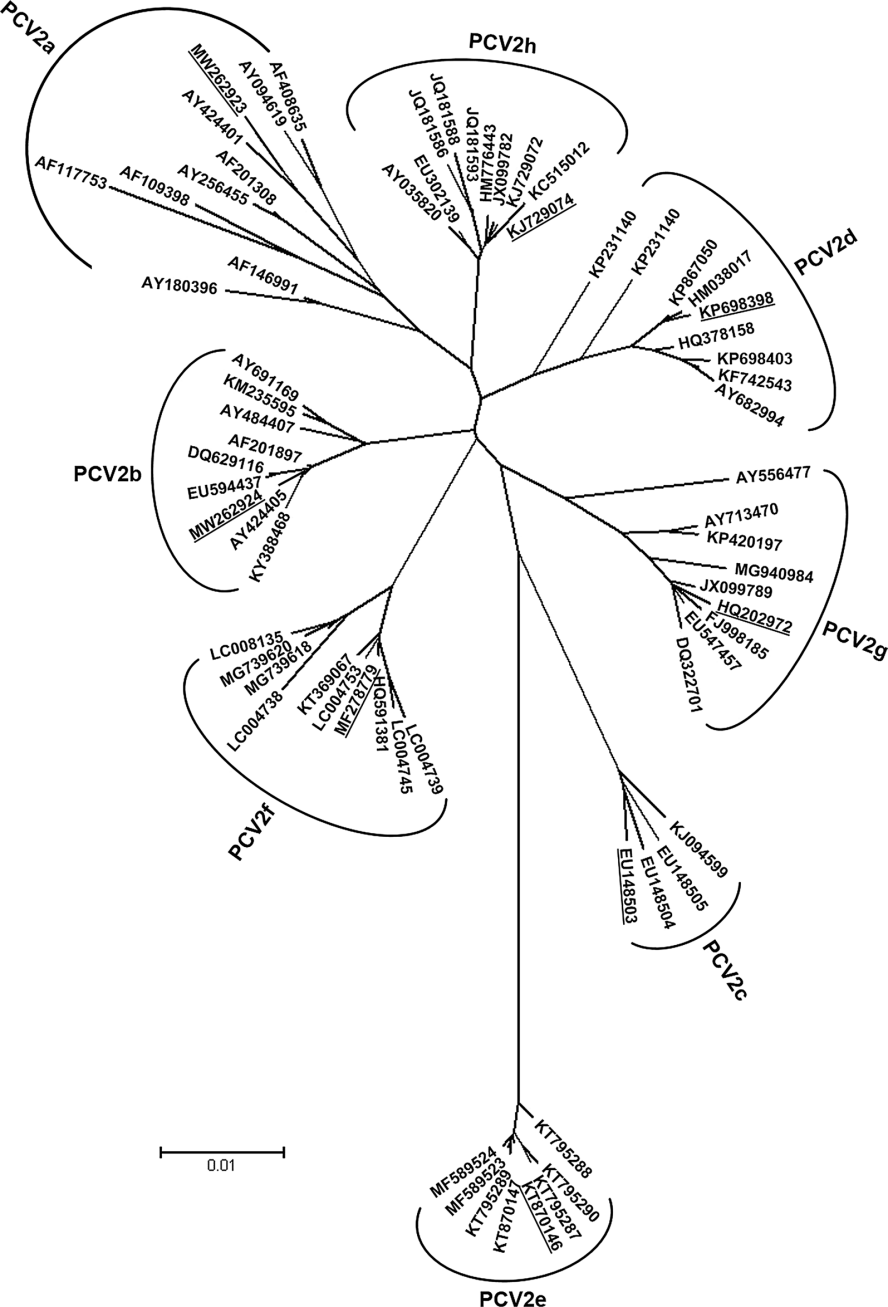
Phylogenetic analysis of PCV2 based on the complete genome of selected PCV2 strains representing the heterogeneity of the eight PCV2 genotypes. The tree was constructed using the neighbour joining method (P-distance model; 1000 bootstraps). The scale bar indicates nucleotide substitutions per site. Underlined reference sequences were used for the evaluation of the PCV2 genotype specific qPCR assays

Reference genomes for the eight PCV2 genotypes were selected according the analysis of Franzo and Segales [4] (Fig. 1). For PCV2c, PCV2e, PCV2f and PCV2g all availlable full genome sequences were analysed, and supplemented by available ORF2 sequences, if necessary to cover the heterogeneity within a genotype. Due to the large number of availlable sequences for PCV2a, PCV2b, PCV2d and PCV2h, we selected up to 100 sequences per genotype, representing the full diversity of each group (e.g. all PCV2a subtypes, according to [13].

**Fig. 2:**
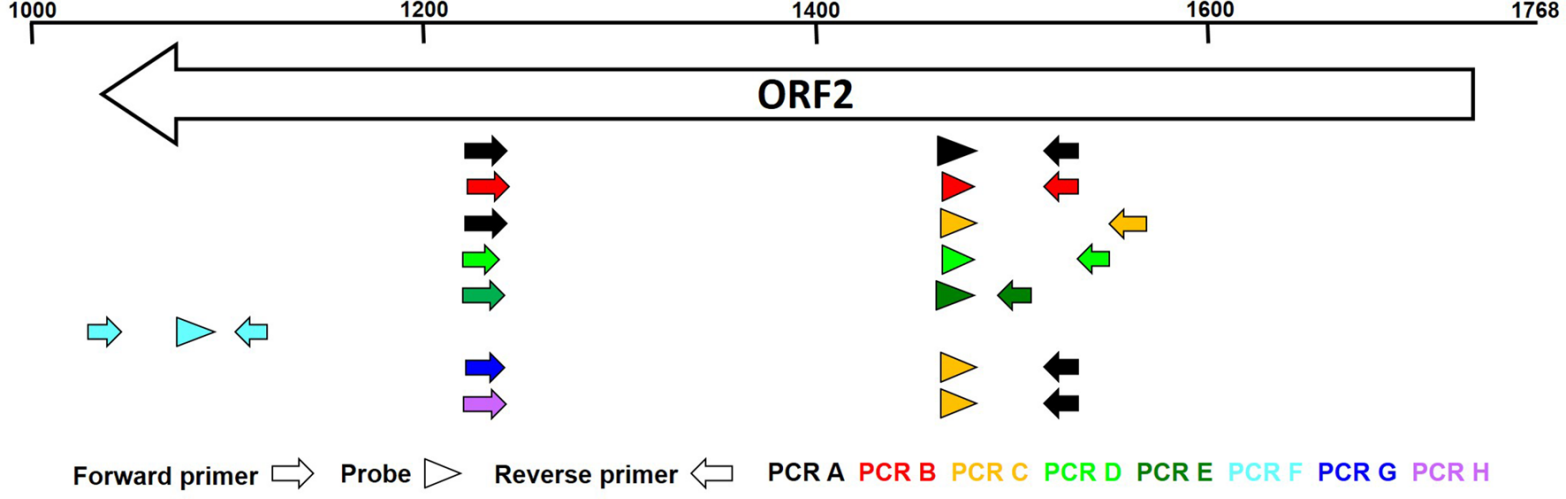
Position of the oligonucleotide primers and TaqMan probes of the eight genotype specific qPCR assays (A-H), relative to the PCV2a genome (upper scale) and ORF2. Primers of PCR A were used in PCR C, G and H, too. For PCR C, G and H the identical TaqMan probe was used

**Fig. 3:**
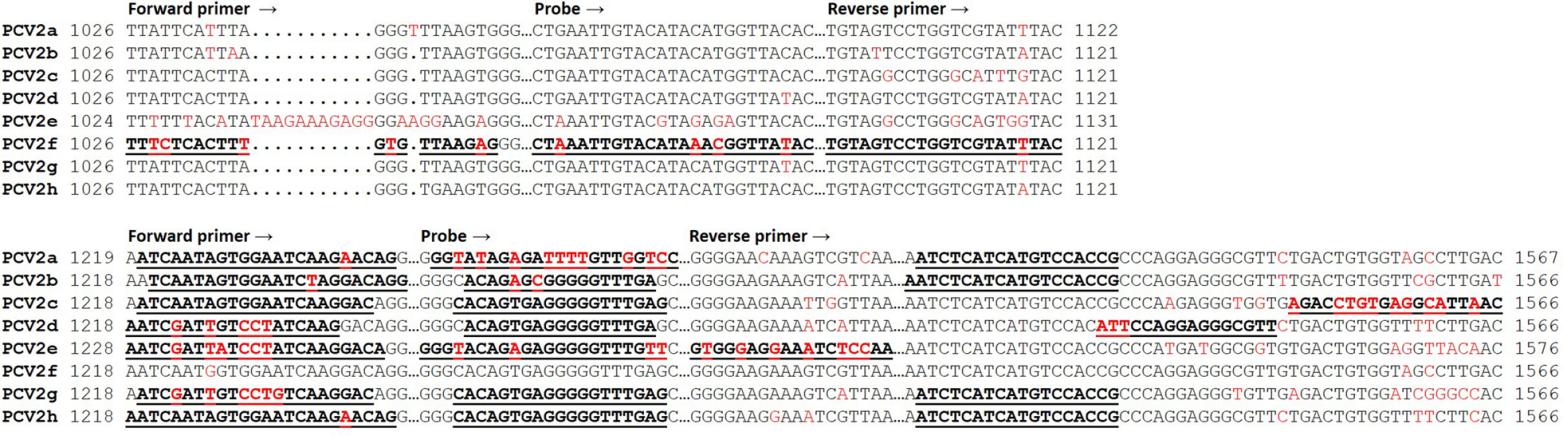
Binding sites of oligonucleotide primers and TaqMan probes. Consensus sequences of the PCV2 genotypes (with indicated position in the full genome) show the binding position of the used oligonucleotide primer or probes (bold and underlined). Letters in red demonstrate motifs discriminating the PCV2 genotypes

After alignment of the sequences using DNASTAR software, we evaluated consensus sequences or SNPs (single nucleotide polymorphism) specific for PCV2 genotypes, respectively. When possible, oligonucleotide primer and TaqMan probes were designed to be specific for only one genotype. However, some primers or probes were used in several PCR assays. In some cases we designed more primers for one genotype to cover the heterogeneity within one genotype and to avoid the use of too many degenerate bases. Additionaly, we used locked nucleic acid (LNA) technology to increase the specific binding of oligonucleotides. Overall, we designed eight singleplex TaqMan-PCRs, six (PCR A-F) specific for only one genotype (PCV2a-PCV2f) and two assays (PCR G and H) detecting two genotypes (PCV2g/PCV2d and PCV2h/PCVc) simultaneously (Figs. 2, 3, Tables 2, 3). All primers and TaqMan probes (inluding LNA modified oligonucleotides) were obtained from Merck (Sigma-Aldrich Chemie GmbH, Germany).

**Table 2:** Oligonucleotide primers and TaqMan probes used for specific detection of PCV2 genotypes

Primer/probe	Sequence 5′–3′	Length
PCV2a_V1-F	ATC AAT AGT GGA RTC RAG AAC AG	23
PCV2a_V2-F	ATC AAT RGT GGA ATC AAG GAC	21
PCV2a-R	CGG TGG ACA TGM TGA GAT	18
PCV2b-F	TCA ATA GTG GAA TCT AGG ACA GG	23
PCV2b-R	CGG TKG ACA TGM TGA GAT T	19
PCV2c-R	GTT AAT GCC TCA CAG GTC T	19
PCV2d-F	AAT CGA TTG T**C**C YAT CAA G	19
PCV2d-R	AAC GCC CTC CTG GAA T	16
PCV2e-F	AAT CGA TTA TCC TAT CAA GGA CA	23
PCV2e-R	TTG GAG ATT TCC TCC CAC	18
PCV2f-F	TTT **C**TC ACT TTG **T**GT TAA G**A**G	21
PCV2f-R	GTA **A**AT ACG ACC AGG ACT ACA	21
PCV2g_V1-F	CGA TTG TCC TGT CAA GGA C	19
PCV2g_V2-F	ATC GAT TGT CCT ATC AAG GA	20
PCV2h_V1-F	AAT CAA TAG TGG AAT CAA GAA CAG	24
PCV2h_V2-F	ATC GAT AGT GGA ATC AAG AAC AG	23
Probe PCV2a	FAM- GGT ATA GAG ATT **T**TG TT**G** GT**C** C -BHQ1	22
Probe PCV2b	FAM- ACA G**A**G **C**GG GGG TTT GA -BHQ1	17
Probe PCV2cgh	FAM- CAC AG**T** G**A**G GGG GTT TGA G -BHQ1	19
Probe PCV2d	FAM- ACA G**T**G **A**GG GGG TTT GA -BHQ1	17
Probe PCV2e	FAM- GGG TAC AGA GAG GGG GTT TGT **T** -BHQ1	22
Probe PCV2f	FAM- CT**A** AAT TGT ACA TA**A** ACG GTT A**T**A C -BHQ1	25

Bold and underlined: nucleotide with LNA modification

### PCV2 genotype specific TaqMan PCR assays

For real-time PCR we used the Luna Universal Probe qPCR (New England BioLabs). 10 µl of the master mix, 4.4 µl RNase free water, 1.6 µl genotype specific primer–probe mix (Table 3) and 4 µl DNA template yielded a final volume of 20 µl per reaction. The thermal profile of the PCR was: 95 °C for 1 min, and 40 cycles of 95 °C for 15 s, 59 °C for 20 s and 68 °C for 20 s. We used the AriaMx real-time PCR system (Agilent) and the corresponding Aria 1.7 software to perform and analyze the qPCRs. Experiments with serial dilutions of the templates were repeated in two independent runs.

### PCV2 genotype specific DNA for testing PCR efficiency and specificity

To evaluate the eight PCV2 genotype specific qPCRs, we used reference or consensus sequences of each PCV2 genotype as target. PCV2a (GenBank MW262923), PCV2b (MW262924) and PCV2d (KP698398) full genome sequences were obtained from isolated virus strains originally obtained from German diagnostic samples in 2013 and 2014, and regularly passaged on PK15 cells. Because virus strains of the other genotypes were not available, we used synthesized DNA covering the target regions with the sequences of the following reference strains: PCV2c: EU148503, PCV2e: KT870146, PCV2f: MF278779, PCV2g: HQ202972 and PCV2h: KJ729074. Log10 dilutions of the templates were tested in duplicates, respectively. Amplification efficiency was calculated the following: *E* = − 1 + 10^(−1/slope)^ [43].

### Testing of diagnostic sensitivity

To estimate the diagnostic sensitivity of the PCV2a and PCV2d specific genotype qPCRs, we used oral fluid samples obtained from mainly subclinically infected pigs as part of an epidemiological study (approved by the ethic committee of the Centre for Clinical Veterinary Medicine, LMU Munich, approval number 191-05-11-2019) performed in Germany in 2019 (manuscript in preparation). Samples containing only one PCV2 genotype (22 PCV2a; 34 PCV2d) were tested with a recently described pan-PCV2 qPCR serving as standard assay for detection and quantification of PCV2 [37, 44, 45], and the genotype specific qPCRs, respectively.

## Results

### Design of PCV2 genotype specific oligonucleotide primers and TaqMan probes

In order to enable genotype specific detection using qPCR, suitable target regions in the PCV2 genome had to be identified that have a high inter-genotype variety and a high degree intra-genotype conservation. Moreover, these regions had to cumulate closely together, to allow fast and efficient amplification with PCR. Therefore, the designed oligonucleotides (excluding PCR F) targeted only binding sites within ORF2 (Fig. 2). Nucleotide position 1219–1243 (refering to PCV2a full genome) was used as binding site for the forward primers (excluding PCR F), because it was recently described that genotype PCV2d significantly differs from PCV2a and PCV2b in this region [23]. The same was observed for PCV2e and PCV2g. The binding sites of the TaqMan probes were between nucleotide 1460–1482, a region that was already targeted by the duplex qPCR described by Opriessnig and colleagues [42]. The reverse primers were targeting a region between nucleotide 1492–1567, in order to complete genotype specific forward pimers/TaqMan probes (PCRs A, B, G and H) or to add another genotype specific element (PCRs C, D and E). The size of the amplified PCR products varied between 291 bp (PCR E) and 337 bp (PCR C). Because we were not able to design a sensitive and specific qPCR for genotype PCV2f at this location of the PCV2 genome, we used distinguishing sequences at the C-terminal end of ORF2, alternatively. Here, a PCV2f specific fragment of 97 bp was amplified.

### Efficiency of the PCV2 genotype specific qPCRs

We tested log10 dilution series of PCV2 genotype specific templates in duplicates to evaluate the amplification efficiency of the eight qPCR assays. All qPCRs demonstrated sufficient efficiency > 90%, however, significant differences were observed. PCR E, F and H performed best with mean efficiency values > 97%, followed by PCR B, C, G (93–96%) and PCR A and D (91–92%).

### Diagnostic sensitivity of the PCV2 genotype specific qPCRs

**Fig. 4:**
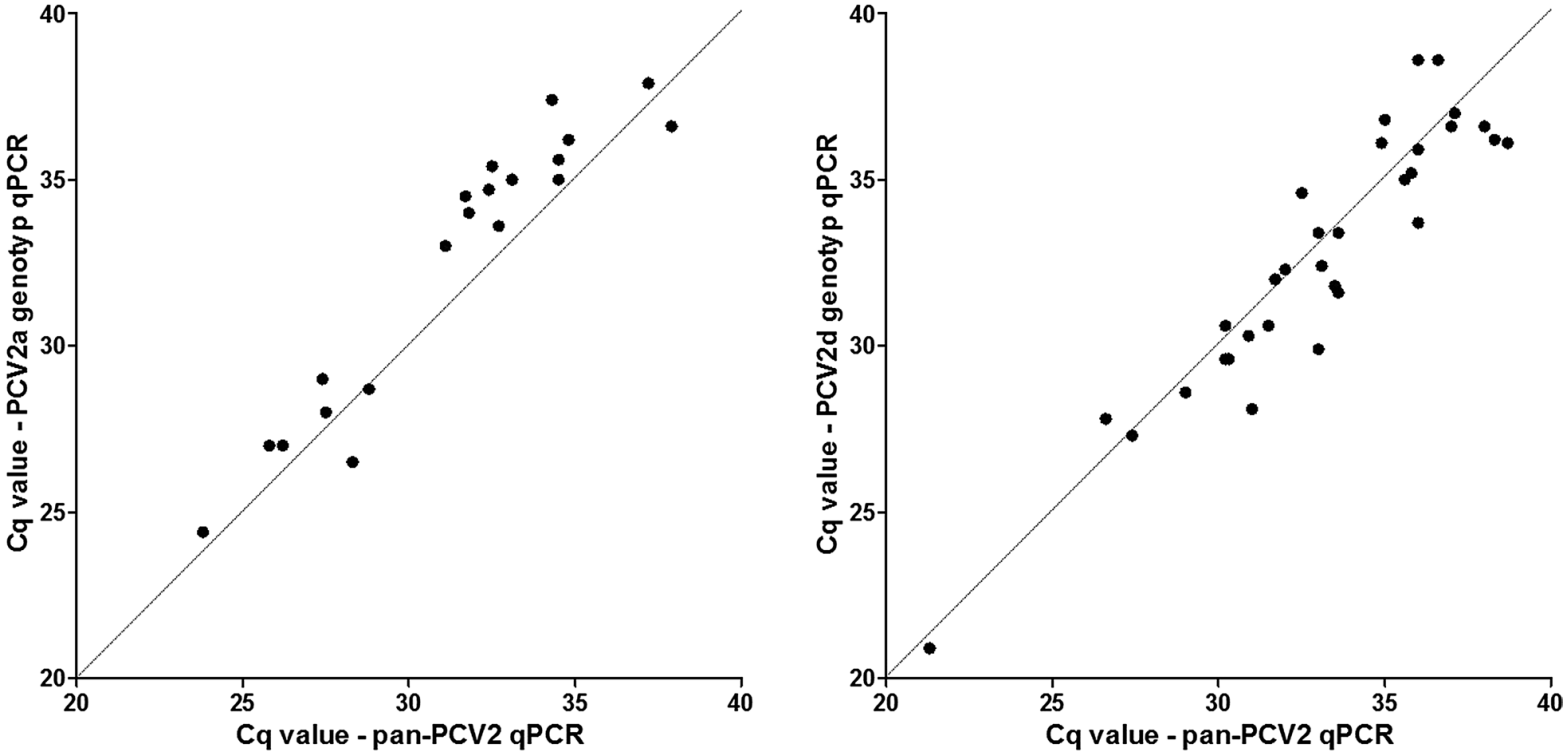
Comparison of PCV2a (left panel) and PCV2d (right panel) genotype specific qPCR with our standard pan-PCV2 qPCR. Shown are the Cq values of diagnostic samples (oral fluids) from pigs infected with PCV2a or PCV2d, respectively

The idea behind our qPCR system was to simplify PCV2 genotyping and not to use these assays for primary PCV2 diagnostics or quantification of viral loads. Nevertheless, a high diagnostic sensitivity of the qPCRs is required, to be a useful tool for investigation of samples with low virus loads, e.g. from subclinically infected animals. Therefore, we compared PCR A and D, which demonstrated lowest efficiency values, with our standard pan-PCV2 qPCR (Fig. 4). We use the latter (estimated limit of detection: 10–20 genome copies per reaction) for diagnostics as well as for epidemiological studies to detect and quantify PCV2 positive samples. For this purpose, we used oral fluid samples from German farms containing different amounts of PCV2a (22 samples; Cq values of the pan-PCV2 qPCR 23.8 to 37.9) or PCV2d (34 samples, Cq values 21.3 to 38.7), respectively. On average, Cq values in PCR A were 1.2 higher compared to the pan-PCV2 qPCR, with a maximum deviation of 3.1, whereas PCR D resulted 0.4 lower Cq values.

### Specificity of the PCV2 genotype qPCRs

**Fig. 5:**
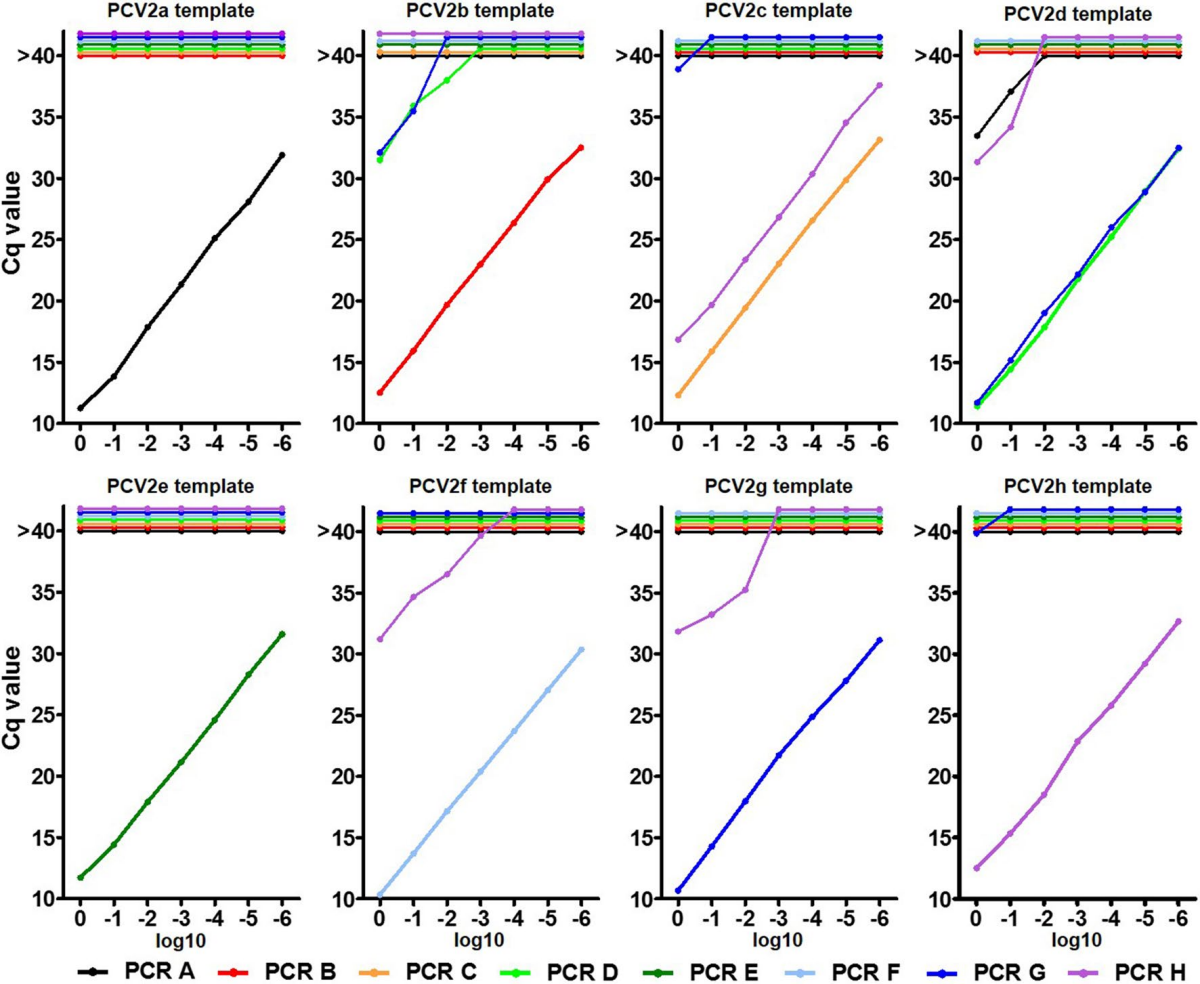
Standard curves of PCV2 genotype specific PCR assays (A-H) based on serial log10 dilutions of genotype specific DNA template. Mean Cq values of two independent experiments

To evaluate the specificity of PCR A-H, we tested dilution series (in a clinically relevant range, starting with Cq values of 10–12) of all genotype templates with every PCR system (Fig. 5). qPCRs B, C, E and F (detecting the corresponding PCV2 genotype) showed no unspecific signals, when DNA sequences of other genotypes were used as template. qPCR A (target PCV2a) showed amplification curves, when high concentrations of the PCV2d template were tested. Here, mean Cq values of 33.5 (undiluted PCV2d template, which resulted Cq 11.4 in the PCV2d-specific qPCR) and 37.1 (1:10 dilution of the PCV2d template, which resulted Cq 14.5 in the PCV2d-specific qPCR) were obtained. That means the difference between the nonspecific (PCR A) and the specific (PCV D) signal was more than 20 Cq values. Likewise, qPCR D (target PCV2d) and PCR G (target PCV2d and PCV2g) showed weak unspecific signals when high amounts of PCV2b template were tested. qPCR H (targeting genotypes PCV2h and PCV2c) demonstrated limited cross reactions with template PCV2d, PCV2f and PCV2g. However, in all these cases the distances between specific and unspecific signals were between 17.3 and 21.2 Cq values.

## Discussion

Because of the considerable increase in the number of available genome sequences worldwide and the high mutation and recombination rate of PCV2, regular revision of existing intra-species classification schemes is important. Recently, a new PCV2 genotyping methodology based on the capsid protein gene (ORF2) divided the heterogeneous population of PCV2 strains into eight genotypes (PCV2a to PCV2h) [4]. However, in order for this knowledge to be properly used in the scientific and veterinary community, feasible genotyping methods must be available. These have to account for complex or theoretical studies (e.g. epidemiological studies on associated diseases or vaccine mediated cross protection) but also practical issues (like routine testing in diagnostic settings) [2]. Therefore, rapid and reliable implementation and practicability are important features of a new test system. For routine diagnostics, a typical workflow for PCV2 genotyping includes PCV2 detection using qPCR, amplification of larger genome fragments, control and clean-up of the PCR products, sequencing and annotation of sequencing results, and phylogenetic analysis using reference genomes and suitable software programs. For single samples of good quality and experienced operators, this approach is quite feasible. However, limitations of that workflow become evident, in cases of large epidemiological studies with high sample numbers, low quality samples or specimens with low virus load, e.g. subclinical PCV2 infections. Here, additional work time (and costs) for nested PCRs or pre-amplification of circovirus genomes (e.g. using RCA) have to be spend to increase sensitivity [14, 40]. The versatile advantages of real-time PCR technology in virology and diagnostics are well established [46–49]. A most significant improvement is the rapidity with which qPCR can produce results. This advantage was utilized by various groups for genotyping of several virus species [50–53], including PCV2 [41, 42]. Many of these assays use primers able to bind at conserved regions of the viral genome in combination with probes (labeled with different fluorophores), specific for genotype discriminating sequences. Moreover, this approach allows multiplex PCR. However, concerning the increasing number of PCV2 genotypes, the heterogeneity within some of the PCV2 genotypes (e.g. PCV2a) and the wide distribution of genotype specific sequence pattern or SNPs within ORF2, we were not able to design such an assay. To grant high specificity and sensitivity of the method, we designed eight singleplex qPCRs, specific for one (PCR A-F) or two (PCR G and H) PCV2 genotypes. For example, PCR G detects genotype PCV2g as well as PCV2d. Both genotypes share similar sequence features, which is why many members of genotype PCV2g were denominated as PCV2d-1 in older classification schemes. To ensure the high sensitivity of the qPCR, we did not enhance the specificity for PCV2g, but designed a second qPCR (PCR D) specific only for PCV2d in order to rule out PCV2g infection. Since PCV2g is of minor interest for swine industry worldwide, this is a very practical approach. Overall, this allows for efficient combination of PCR tests, regarding to the diagnostic task and the epidemiological background. In German swine industry, for example, the genotypes PCV2a, 2b and 2d are prevalent in cases of PCVD [14, 23, 37]. Therefore, our workflow in clinical cases is the following: detection and quantification of PCV2 with a pan-PCV2 reactive qPCR [44] and afterwards genotyping using PCR A, B and D. If Cq values of the pan-PCV2 qPCR correspond with the result of one of the genotype specific qPCRs, PCVD diagnosis is completed. If not (significantly lower Cq values in the pan-PCV2 qPCR), the remaining genotype specific PCRs will be applied. In this way, the introduction of a new PCV2 genotype can be easily detected. In other regions of the world, the setup might be adapted, e.g. in North America PCR E might become a part of the start setting or if European wild boars are tested, PCR G should be included.

Single unique primers or probes or the tactical combination of oligonucleotides generate the PCV2 genotype specificity of the several PCRs (Fig. 2, Table 3). In some cases, however, only a few genotype specific SNPs were available that we could use as targets for discriminating primers or probes. Therefore, we tried to enhance the relevance of these positions by using locked nucleic acid technology. A LNA monomer is a 2′-*O*,4′-C-methylene-linked ribonucleotide with a locked C3′-endo sugar conformation, which significantly increases hybridization properties (e.g. melting temperature, duplex stability and specificity). Therefore, LNA is a versatile tool e.g. for SNP detection [54, 55]. Using this technology, we were able to design shorter oligonucleotides with enhanced affinity for the genotype specific sequences.

**Table 3:** Composition and specificity of the primer–probe (PP) mixes

PP	Components (concentration in PCR mix)	Specificity
A	PCV2a_V1-F (400 nM) + PCV2a_V2-F (400 nM) + PCV2a-R (800 nM) + probe PCV2a (200 nM)	PCV2a
B	PCV2b-F (800 nM) + PCV2b-R (800 nM) + probe PCV2b (200 nM)	PCV2b
C	PCV2a_V2-F (800 nM) + PCV2c-R (800 nM) + probe PCV2cgh (200 nM)	PCV2c
D	PCV2d-F (800 nM) + PCV2d-R (800 nM) + probe PCV2d (200 nM)	PCV2d
E	PCV2e-F (800 nM) + PCV2e-R (800 nM) + probe PCV2e (200 nM)	PCV2e
F	PCV2f-F (800 nM) + PCV2f-R (800 nM) + probe PCV2f (200 nM)	PCV2f
G	PCV2g_V1-F (400 nM) + PCV2g_V2-F (400 nM) + PCV2a-R (800 nM) + probe PCV2cgh (200 nM)	PCV2g/2d
H	PCV2h_V1-F (400 nM) + PCV2h_V2-F (400 nM) + PCV2a-R (800 nM) + probe PCV2cgh (200 nM)	PCV2h/2c

Nevertheless, in a limited number of the PCV2 genotype specific PCRs (especially PCR H) slightly unspecific signals were obtained, when a very high copy number of some non-targeted genotypes was tested. In severe cases of PCVD such high viral loads may appear in routine diagnostics, however, distances between specific and unspecific signals were about 20 Cq values (equivalent to about a one million-fold difference). In consequence, the operator must interpret such test results carefully in direct context with the results from the other qPCRs. For example in abortion samples with a Cq value of 15 for PCV2d, a Cq value of 37 for PCV2a might just be ignored, as it might not be etiological for the clinical outcome. Otherwise, in our experience, randomly collected samples for epidemiological PCV2 studies rarely demonstrate viral loads to allow these unspecific signals to occur.

A major advantage of our genotyping system is the ability to easily detect multiple PCV2 genotypes within one sample. Coinfections of single animals with different PCV2 strains or genotypes and recombination events have been reported since the rise of PCV2 as relevant pathogen [3, 36, 56–58]. In cases with comparable virus loads of the different virus strains, this may cause low quality sequencing results and cloning of the PCR products might be necessary in order to obtain readable sequences [57]. Due to own observations, mixed sequences of insufficient quality can sometimes be misinterpreted as recombinants or “new” virus variants. On the other hand, in samples with uneven virus loads of different genotypes, it is likely that only the dominant virus is detected and sequenced probably, if standard procedures were used. This might significantly bias epidemiological studies about distribution of genotypes in the field.

We designed our PCR system as a kind of “second-line” diagnostic tool for easy and specific genotyping and not for primary PCV2 diagnostics. For PCV2 detection (demanding a maximum in sensitivity) and quantification of virus loads well-established pan-PCV2 qPCRs are available and continue to be useful. Therefore, we have refrained from determining the analytical sensitivity, the limit of detection or the limit of quantification of the eight PCV2 genotype specific PCRs. Instead, we choose a very practical approach to investigate the diagnostic sensitivity of the PCR A and D, which demonstrated the lowest PCR efficiency in dilution experiments. We used oral fluid samples collected and processed within a highly standardized study about the occurrence of PCV2 genotypes in Germany (manuscript in preparation) and compared results of the relevant PCV2 genotype specific PCRs with our recently described standard pan-PCV2 qPCR (Fig. 4). Since no relevant differences were obtained and samples with low virus load and Cq values above 35 could be clearly assigned to genotype PCV2a or PCV2d, respectively, we concluded that the diagnostic sensitivity of these PCV2 genotype specific qPCRs is sufficient to reliably identify PCV2 genotypes in samples from pigs with subclinical PCV2 infection.

In diagnostics, the robustness of a qPCR is an important quality characteristic, too. Minor deviations in annealing temperature or time, in concentrations of primers or probes, in salt concentrations of the used PCR chemistry, or the use of different polymerases or real time PCR machines should not affect the outcome of the analysis. For the introduced genotyping PCR system, however, we must point out that even small changes in the setting can negatively affect specificity or sensitivity. For example, in pre-experiments (data not shown), we compared different qPCR kits of several suppliers. A higher level of unspecific signals was obtained when the Luna Universal Probe qPCR chemistry was not used. It might be speculated, if an adjustment of the annealing temperature would have resolved this problem. However, if annealing temperature was increased slightly from 59 to 60 °C in our setting, efficiency and sensitivity of some of the assays decreased in an unacceptable manner (data not shown).

Olvera and colleagues demonstrated early that analysis of ORF2 is sufficient to investigate phylogenetic relationships between PCV2 strains [13]. Therefore, also the updated PCV2 genotyping methodology by Franzo and Segales is based on ORF2 sequences [4]. In agreement with these data, our genome analysis also revealed sequences located in ORF2 to be prime targets for genotyping with qPCR. However, analyzing only fragments or a single ORF of the viral genome always has the disadvantage, that e.g. recombination events could be missed. The dataset that was analyzed for the phylogeny-based genotype definition, obtained more than 4500 ORF2 sequences. More than 99% of these were classified as members of one of the eight defined genotypes (PCV2a to PCV2h). However, some of the remaining sequences also formed phylogenetic clusters, but were not recognized as new PCV2 genotypes due to the limited number of available sequences and to avoid the risk of defining poor quality sequences as separate genotypes [4]. We excluded those sequences from our analysis, too. Therefore, the ongoing evolution of these or other PCV2 strains has to be monitored constantly. Our evaluation of the PCV2 genotype specific qPCRs used a limited number of sequences, which cannot cover the complete phylogenetic diversity of PCV2. Therefore, future adaptations of the described PCV2 genotyping PCR system might be necessary and the ongoing sequencing of PCV2 strains and provision of this information in freely available databases remains of high importance. We would like to emphasize that we consider our PCV2 genotyping PCR system to be a valuable and helpful tool that complements standard sequencing methods.

## Conclusion

Genotyping of PCV2 is important for routine diagnosis as well as for scientific studies. Typically, sequencing of the full circovirus genome or at least the ORF2 sequence is needed for genotype assignment. Disadvantages of this method are the missing mass compatibility, limited speed and sensitivity. Therefore, we designed and evaluated a fast, sensitive and reliable genotyping system based on eight singleplex TaqMan real-time PCRs. This system might be a valuable complement to the classical sequencing methodology, especially in cases of simultaneous infections with multiple PCV2 genotypes, subclinically infected animals or epidemiological studies with large sample numbers.

## Data Availability

Not applicable.

## Abbreviations

aa: Amino acid(s)
bp: Base pair(s)
Cq: Quantification cycle
DNA: Deoxyribonucleic acid
LNA: Locked nucleic acid
nM: Nanomolar
nn: Nucleotide(s)
ORF: Open reading frame
PCR: Polymerase chain reaction
PCV: Porcine circoviruses
PCVD: PCV2 associated disease
PCV2: Porcine circovirus type 2
PMWS: Postweaning multisystemic wasting syndrome
qPCR: Quantitative PCR
RCA: Rolling circle amplification
SNP: Single nucleotide polymorphism

## References

[1] Firth C, Charleston MA, Duffy S, Shapiro B, Holmes EC (2009). Insights into the evolutionary history of an emerging livestock pathogen: porcine circovirus 2. J Virol.

[2] Franzo G, Cortey M, Olvera A, Novosel D, De Castro AMMG, Biagini P (2015). Revisiting the taxonomical classification of Porcine Circovirus type 2 (PCV2): Still a real challenge. Virol J.

[3] Franzo G, Cortey M, Segalés J, Hughes J, Drigo M (2016). Phylodynamic analysis of porcine circovirus type 2 reveals global waves of emerging genotypes and the circulation of recombinant forms. Mol Phylogenet Evol.

[4] Franzo G, Segalés J (2018). Porcine circovirus 2 (PCV-2) genotype update and proposal of a new genotyping methodology. PLoS ONE.

[5] Jacobsen B, Krueger L, Seeliger F, Bruegmann M, Segalés J, Baumgaertner W (2009). Retrospective study on the occurrence of porcine circovirus 2 infection and associated entities in Northern Germany. Vet Microbiol.

[6] Allan GM, McNeilly F, Kennedy S, Daft B, Clarke EG, Ellis JA (1998). Isolation of porcine circovirus-like viruses from pigs with a wasting disease in the USA and Europe. J Vet Diagn Invest.

[7] Allan G, Krakowka S, Ellis J, Charreyre C (2012). Discovery and evolving history of two genetically related but phenotypically different viruses, porcine circoviruses 1 and 2. Virus Res.

[8] Dupont K, Nielsen EO, Bækbo P, Larsen LE (2008). Genomic analysis of PCV2 isolates from Danish archives and a current PMWS case–control study supports a shift in genotypes with time. Vet Microbiol.

[9] Hamel AL, Lin LL, Nayar GPS (1998). Nucleotide sequence of porcine circovirus associated with postweaning multisystemic wasting syndrome in pigs. J Virol.

[10] Segalés J, Olvera A, Grau-Roma L, Charreyre C, Nauwynck H, Larsen L (2008). PCV-2 genotype definition and nomenclature. Vet Rec.

[11] Cheung AK, Lager KM, Kohutyuk OI, Vincent AL, Henry SC, Baker RB (2007). Detection of two porcine circovirus type 2 genotypic groups in United States swine herds. Arch Virol.

[12] Gagnon CA, Tremblay D, Tijssen P, Venne M-H, Houde A, Mehdy ES (2007). Article the emergence of porcine circovirus 2b genotype (PCV-2b) in swine in Canada. CVJ.

[13] Olvera A, Cortey M, Segalés J (2007). Molecular evolution of porcine circovirus type 2 genomes: phylogeny and clonality. Virology.

[14] Reiner G, Hofmeister R, Willems H (2015). Genetic variability of porcine circovirus 2 (PCV2) field isolates from vaccinated and non-vaccinated pig herds in Germany. Vet Microbiol.

[15] Weissenbacher-Lang C, Kristen T, Mendel V, Brunthaler R, Schwarz L, Weissenböck H. Porcine circovirus type 2 (PCV2) genotyping in Austrian pigs in the years 2002 to 2017. BMC Vet Res. 2020;16. 10.1186/s12917-020-02413-4 PMC7294622 32539835

[16] Wiederkehr DD, Sydler T, Buergi E, Haessig M, Zimmermann D, Pospischil A (2009). A new emerging genotype subgroup within PCV-2b dominates the PMWS epizooty in Switzerland. Vet Microbiol.

[17] Franzo G, Cortey M, de Castro AMMG, Piovezan U, Szabo MPJ, Drigo M (2015). Genetic characterisation of Porcine circovirus type 2 (PCV2) strains from feral pigs in the Brazilian Pantanal: an opportunity to reconstruct the history of PCV2 evolution. Vet Microbiol.

[18] Liu X, Wang FX, Zhu HW, Sun N, Wu H (2016). Phylogenetic analysis of porcine circovirus type 2 (PCV2) isolates from China with high homology to PCV2c. Arch Virol.

[19] Guo LJ, Lu YH, Wei YW, Huang LP, Liu CM (2010). Porcine circovirus type 2 (PCV2): genetic variation and newly emerging genotypes in China. Virol J.

[20] Guo L, Fu Y, Wang Y, Lu Y, Wei Y, Tang Q (2012). A porcine circovirus type 2 (PCV2) mutant with 234 amino acids in Capsid protein showed more virulence in vivo, compared with classical PCV2a/b strain. PLoS ONE.

[21] Opriessnig T, Xiao CT, Gerber PF, Halbur PG, Matzinger SR, Meng XJ (2014). Mutant USA strain of porcine circovirus type 2 (mPCV2) exhibits similar virulence to the classical PCV2a and PCV2b strains in caesarean-derived, colostrum-deprived pigs. J Gen Virol.

[22] Opriessnig T, Xiao CT, Gerber PF, Halbur PG. Emergence of a novel mutant PCV2b variant associated with clinical PCVAD in two vaccinated pig farms in the U.S. concurrently infected with PPV2. Vet Microbiol. 2013;163:177–83. 10.1016/j.vetmic.2012.12.019 23305615

[23] Eddicks M, Fux R, Szikora F, Eddicks L, Majzoub-Altweck M, Hermanns W (2015). Detection of a new cluster of porcine circovirus type 2b strains in domestic pigs in Germany. Vet Microbiol.

[24] Xiao CT, Halbur PG, Opriessnig T (2015). Global molecular genetic analysis of porcine circovirus type 2 (PCV2) sequences confirms the presence of four main PCV2 genotypes and reveals a rapid increase of PCV2d. J Gen Virol.

[25] Grierson SS, King DP, Wellenberg GJ, Banks M (2004). Genome sequence analysis of 10 Dutch porcine circovirus type 2 (PCV-2) isolates from a PMWS case–control study. Res Vet Sci.

[26] Wei R, Xie J, Theuns S, Nauwynck HJ. Changes on the viral capsid surface during the evolution of porcine circovirus type 2 (PCV2) from 2009 till 2018 may lead to a better receptor binding. Virus Evol. 2019;5:vez026. 10.1093/ve/vez026 PMC6676070 31392030

[27] Xiao CT, Harmon KM, Halbur PG, Opriessnig T. PCV2d-2 is the predominant type of PCV2 DNA in pig samples collected in the US during 2014–2016. Vet Microbiol. 2016;197:72–7. 10.1016/j.vetmic.2016.11.009 27938686

[28] Davies B, Wang X, Dvorak CM, Marthaler D, Murtaugh MP (2016). Diagnostic phylogenetics reveals a new Porcine circovirus 2 cluster. Virus Res.

[29] Liu J, Wei C, Dai A, Lin Z, Fan K, Fan J (2018). Detection of PCV2e strains in Southeast China. PeerJ.

[30] Bao F, Mi S, Luo Q, Guo H, Tu C, Zhu G (2018). Retrospective study of porcine circovirus type 2 infection reveals a novel genotype PCV2f. Transbound Emerg Dis.

[31] Cadar D, Cságola A, Lorincz M, Tombácz K, Spînu M, Tuboly T (2012). Detection of natural inter- and intra-genotype recombination events revealed by cap gene analysis and decreasing prevalence of PCV2 in wild boars. Infect Genet Evol.

[32] Knell S, Willems H, Hertrampf B, Reiner G (2005). Comparative genetic characterization of Porcine Circovirus type 2 samples from German wild boar populations. Vet Microbiol.

[33] Savic B, Milicevic V, Jakic-Dimic D, Bojkovski J, Prodanovic R, Kureljusic B (2012). Genetic characterization and phylogenetic analysis of porcine circovirus type 2 (PCV2) in Serbia. Arch Virol.

[34] Anoopraj R, Rajkhowa TK, Cherian S, Arya RS, Tomar N, Gupta A (2015). Genetic characterisation and phylogenetic analysis of PCV2 isolates from India: Indications for emergence of natural inter-genotypic recombinants. Infect Genet Evol.

[35] Cai L, Ni J, Xia Y, Zi Z, Ning K, Qiu P (2012). Identification of an emerging recombinant cluster in porcine circovirus type 2. Virus Res.

[36] Grau-Roma L, Crisci E, Sibila M, López-Soria S, Nofrarias M, Cortey M (2008). A proposal on porcine circovirus type 2 (PCV2) genotype definition and their relation with postweaning multisystemic wasting syndrome (PMWS) occurrence. Vet Microbiol.

[37] Eddicks M, Szikora F, Walhöfer N, Louis CS, Reese S, Banholzer E (2017). Vorkommen von Genotypen des Porzinen circovirus Typ 2 (PCV2) in Schweinebeständen mit unterschiedlichen Impfstrategien gegen PCV2. Tierarztl Prax Ausgabe G Grosstiere - Nutztiere.

[38] Karuppannan AK, Opriessnig T (2017). Porcine circovirus type 2 (PCV2) vaccines in the context of current molecular epidemiology. Viruses.

[39] Fenaux M, Halbur PG, Haqshenas G, Royer R, Thomas P, Nawagitgul P (2002). Cloned genomic DNA of Type 2 porcine circovirus is infectious when injected directly into the liver and lymph nodes of pigs: characterization of clinical disease, virus distribution, and pathologic lesions. J Virol.

[40] Fux R, Söckler C, Link EK, Renken C, Krejci R, Sutter G (2018). Full genome characterization of porcine circovirus type 3 isolates reveals the existence of two distinct groups of virus strains. Virol J.

[41] Gagnon CA, Del Castillo JRE, Music N, Fontaine G, Harel J, Tremblay D (2008). Development and use of a multiplex real-time quantitative polymerase chain reaction assay for detection and differentiation of Porcine circovirus-2 genotypes 2a and 2b in an epidemiological survey. J Vet Diagn Invest.

[42] Opriessnig T, Prickett JR, Madson DM, Shen HG, Juhan NM, Pogranichniy RM (2010). Porcine circovirus type 2 (PCV2)-infection and re-inoculation with homologous or heterologous strains: virological, serological, pathological and clinical effects in growing pigs. Vet Res.

[43] Bustin SA, Benes V, Garson JA, Hellemans J, Huggett J, Kubista M (2009). The MIQE guidelines: minimum information for publication of quantitative real-time PCR experiments. Clin Chem.

[44] Eddicks M, Koeppen M, Willi S, Fux R, Reese S, Sutter G (2016). Low prevalence of porcine circovirus type 2 infections in farrowing sows and corresponding pre-suckling piglets in southern German pig farms. Vet Microbiol.

[45] Eddicks M, Beuter B, Stuhldreier R, Nolte T, Reese S, Sutter G (2019). Cross-sectional study on viraemia and shedding of porcine circovirus type 2 in a subclinically infected multiplier sow herd. Vet Rec.

[46] Mackay IM (2004). Real-time PCR in the microbiology laboratory. Clin Microbiol Infect.

[47] Gadkar VJ, Filion M (2014). New developments in quantitative real-time polymerase chain reaction technology. Curr Issues Mol Biol.

[48] Hoffmann B, Beer M, Reid SM, Mertens P, Oura CAL, van Rijn PA (2009). A review of RT-PCR technologies used in veterinary virology and disease control: sensitive and specific diagnosis of five livestock diseases notifiable to the World Organisation for Animal Health. Vet Microbiol.

[49] Kralik P, Ricchi M (2017). A basic guide to real time PCR in microbial diagnostics: definitions, parameters, and everything. Front Microbiol.

[50] Bonvicini F, Manaresi E, Bua G, Venturoli S, Gallinella G (2013). Keeping pace with parvovirus b19 genetic variability: a multiplex genotype-specific quantitative pcr assay. J Clin Microbiol.

[51] Coudray-Meunier C, Fraisse A, Mokhtari C, Martin-Latil S, Roque-Afonso AM, Perelle S (2014). Hepatitis A virus subgenotyping based on RT-qPCR assays. BMC Microbiol.

[52] Schultz AC, Vega E, Dalsgaard A, Christensen LS, Nørrung B, Hoorfar J (2011). Development and evaluation of novel one-step TaqMan realtime RT-PCR assays for the detection and direct genotyping of genogroup I and II noroviruses. J Clin Virol.

[53] Wang X, Guo S, Hameed M, Zhang J, Pang L, Li B (2020). Rapid differential detection of genotype I and III Japanese encephalitis virus from clinical samples by a novel duplex TaqMan probe-based RT-qPCR assay. J Virol Methods.

[54] Petersen M, Wengel J (2003). LNA: A versatile tool for therapeutics and genomics. Trends Biotechnol.

[55] Owczarzy R, You Y, Groth CL, Tataurov AV (2011). Stability and mismatch discrimination of locked nucleic acid-DNA duplexes. Biochemistry.

[56] Correa-Fiz F, Franzo G, Llorens A, Segalés J, Kekarainen T (2018). Porcine circovirus 2 (PCV-2) genetic variability under natural infection scenario reveals a complex network of viral quasispecies. Sci Rep.

[57] de Boisséson C, Béven V, Bigarré L, Thiéry R, Rose N, Eveno E (2004). Molecular characterization of Porcine circovirus type 2 isolates from post-weaning multisystemic wasting syndrome-affected and non-affected pigs. J Gen Virol.

[58] Opriessnig T, McKeown NE, Zhou EM, Meng XJ, Halbur PG (2006). Genetic and experimental comparison of porcine circovirus type 2 (PCV2) isolates from cases with and without PCV2-associated lesions provides evidence for difference in virulence. J Gen Virol.

